# Letting the plants speak: Law, landscape and conservation

**DOI:** 10.1007/s13280-023-01957-7

**Published:** 2023-12-08

**Authors:** Jo Gillespie, Rebecca Hamilton, Dan Penny

**Affiliations:** School of Geosciences, The University of Sydney, Camperdown, NSW 2006 Australia

**Keywords:** Conservation, Legal geography, Palynology, Wetlands

## Abstract

The Botany Wetlands are the contemporary remnant of a formerly extensive coastal freshwater wetland in the inner-urban suburbs of Sydney (Australia). This site supports a range of ecosystem services, including human physical and mental health benefits, filtration of stormwater runoff from a highly urban and industrial catchment, and accommodation space for floodwater. The wetlands also provide habitat to migratory water birds and act as a connective habitat corridor and refuge for native flora and fauna including endangered ecological communities recognised in state and national legislation. Current management strategies and ‘on the ground’ practices are informed by a hierarchy of laws and management plans that act to create and reinforce a specific narrative in the material landscape. Here we consider the ecological history of the wetlands, derived from paleoecological data, in the context of this complex network of governance entanglements. We argue that the system bears little resemblance to its long-term character and has been made and continually re-made by a portmanteau of inflexible regulatory structures. We suggest that maintaining ecosystem services in such a complex, hybridized sociolegal-biophysical system requires a critical view of both the power relations *and* physical processes that shape it.

## Introduction

Restoration of ecosystems modified by human activity rests upon the (often unstated) assumption that some system conditions or states are more desirable than others. Very frequently this devolves to simple binaries—pristine versus degraded, native versus exotic, authentic versus synthetic or hybridised. Such subjective and culturally contingent valuations of environmental condition can become codified into management policy and practices, such that subjective decisions become made and re-made in the material landscape. The Anthropocene concept (Corlett 2015; Bowman et al. 2017) encourages us to recognise that, in the Anthropocene, there are no ‘non-human’ environments—everything and everywhere is, to one degree or another, hybridized (Hobbs et al. 2006; Hobbs 2013). This might be a cause of ‘hopelessness’ in conservation (Caro et al. 2012) or invite scorn from ‘neoliberal postnatural’ conservationists (Collard et al. 2015). However, Clément (2004) Urban (2018) and others challenge us to embrace the promise of penumbral spaces and hybridised systems; to celebrate the “ordinary, vernacular, or degraded” (Urban 2018, p. 51). One reading of this challenge is to avoid becoming entangled in entrenched and largely fruitless debates on the relative merits of alternative but equally subjective environmental narratives, and to embrace a more functionalist approach to the composition and re-composition of ‘degraded’ ecological systems that may be more appropriate for the systemic challenges of the Anthropocene (Cadotte et al. 2011).

While the fundamental assumptions of conservation biology may be morphing in response to rapid and systemic global change (Collard et al. 2015), conservation practice still asserts that some plant and animal communities ‘belong’ while others do not (cf. Carter and Paterson 2021). Gould (1998) makes the point that evolutionary theory upends the notion that organisms were created in place and are therefore perfect, emphasising instead a continuum of adaptations, shifting ranges and speciation. Native plants may be ‘locally appropriate’ but cannot be, according to Darwinian principles, ‘locally optimal’. Time is the confounding factor here—given time, even locally optimal plants and animals will be replaced with others. However, given that environmental managers are hoping to secure urban environments that can sustain ecosystem services through the coming century rather than over the coming millennium, the fleeting nature of local adaptation might be considered practically irrelevant.

Indeed, perfect coherence with Darwinian theory does not resolve the immediate environmental impact of exotic species on habitat and biodiversity, water quality and quantity, soil condition, and so on. Gould (1998) acknowledges this; “at least we know what natives will do in an unchanged habitat, for they have generally been present for a long time and have therefore stabilized and adapted” (p. 9). It is what native species ‘do’ in hybridised urban environments that is of importance to the human and more-than-human communities that share those spaces. This is precisely what Head (2012) concludes—for management, at least, it does not matter what is native or not, but what “behaviours and effects” (p. 174) emerge from hybridised vegetation communities that are important for securing sustainable outcomes. We already know, for example, that heavily modified urban environments contain a higher number of threatened plant and animal species per unit area than non-urban areas and are therefore disproportionally important in biodiversity conservation (Ives et al. 2016). This focus on the functional value of hybrid ecosystems, rather than the ‘authenticity’ of their component parts, allows us to step around alternative and purely subjective environmental narratives to focus on the maintenance or improvement of environmental services that support sustainable futures. This approach emphasises the utilitarian value of nature for humans at the cost of other values, such as intrinsic values (Taylor et al. 2020). We argue that, in the context of spatially restricted blue/green spaces within a heavily urbanised environment that is home to more than 5 million humans, functionalist values should take precedence.

Here, we demonstrate that contemporary conservation practices for high-value inner-urban green and blue spaces are the product of *regulatory* and *institutional* narratives, and do not reflect the ‘authentic’ vegetation that once grew in these areas. We utilise a case study approach that allows us to emphasise locally specific and contextual conditions that are decisive to the outcome while permitting more widely applicable or comparative outcomes to be derived inductively. Our approach deploys palaeo-ecological data derived from the analysis of a sedimentary archive within the context of a dense regulatory filigree that, ultimately, enables or disables particular behaviours and environmental narratives. We then consider the functional value of ‘authentic’ ecosystems and argue that, in some cases at least, the ability to mimic past ecosystems as part of disrupted contemporary urban environments can exploit the self-organising capacity of ecosystems with a long history ‘in place’. We argue that the systemic disruption of the Anthropocene requires urban green spaces *and* governance structures that are able to tolerate increased perturbation without losing previous functional integrity (following Meerow et al.‘s 2016 integrative definition of urban resilience), ensuring the maintenance of environmental services.

## Materials and methods

### Site description, historical and regulatory context

**Fig. 1:**
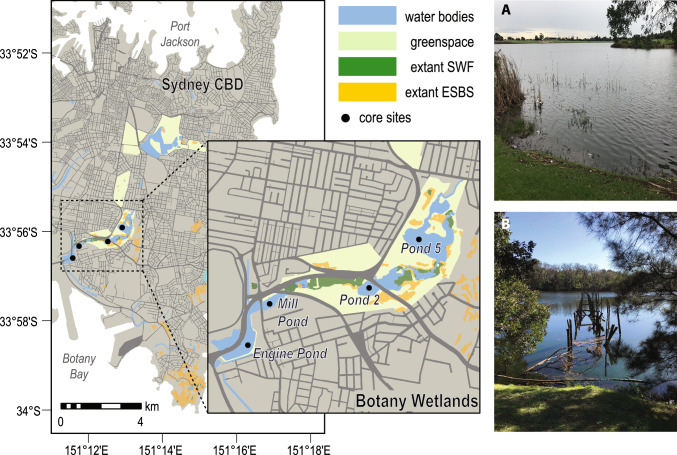
Map of eastern Sydney and the Sydney Central Business District (CBD), New South Wales, Australia, showing the location of the Botany Wetlands ponds within the wetland corridor in relation to the sampling locations (black dots) and the current distribution of Eastern Suburbs Banksia Scrub (ESBS) and Sydney Freshwater Wetland (SFW) Communities. **a** Looking west over Pond 5, a remnant of the pre-European wetlands that formed part of Sydney’s water supply from the mid nineteenth century. **b** Ruins of a jetty that supported a water pump used to wash wool until around 1900, looking north over the Mill Pond east of Botany Road

The Botany Wetlands are the largest coastal freshwater wetlands in the Sydney region, stretching along a 4.5 km corridor that runs through Sydney’s inner eastern suburbs (Fig. 1) on unceded lands of the Gadigal and Dharawal people. Rising from a natural freshwater spring in what is now Centennial Park, the Botany Wetlands was an extensive wetland that followed the drainage line from Randwick to the south-south-west, eventually discharging into Kamay Botany Bay near the present Sydney airport site. The wetland system has been significantly altered since European colonisation as a result of industrialisation and subsequent urbanisation.

The wetland corridor was an important resource for First Nations people prior to European arrival (Benson and Howell 1990; Attenbrow 2010; Booth 2021) and, to some extent, its utility and the Law surrounding its use was shared with some early colonists (Hinkson 2002; Goodall 2017). Despite the undisguised antipathy with which the mangroves and wetlands on the northern shore of Kamay Botany Bay were met by early colonists, the southern end of the Botany Wetlands system was valued for industry and was alienated for private property and used for industrial purposes. Dams were built from 1815, where the wetlands discharged into Kamay Botany Bay, to facilitate aspects of this industrial activity.

The *Sydney Water Supply Act 1833* (NSW) represents the earliest appropriation of land in Australia for the purpose of water supply (Williams 1989; Davies and Wright 2014). The subsequent *Sydney City Incorporation Act 1842* (NSW) empowered the newly formed Sydney City Corporation to manage (*inter alia*) water resources and maintain a water supply. The importance of a stable water supply for the burgeoning town was well understood by this time and in 1855, following a severe drought in 1849 that stimulated a Special Committee of Inquiry, the Sydney City Corporation began resuming land along the wetland corridor to secure potable water for the colony (Aird 1961). The Botany Swamps Water Supply Scheme (BSWSS) was intended to augment the existing water abstraction from the system via Busby’s Bore, established nearly two decades earlier, which exploited the natural spring at Lachlan Swamp at the top of the catchment, but which was insufficient to meet demand (Obateru 1971). The BSWSS began operation in 1859 under the control of the newly formed Sydney City Council but was also unable to meet demand, and in 1866–1867 the capacity of the wetlands was increased by the creation of six dams along the drainage line, effectively fragmenting the floodplain into a series of discrete waterbodies.

Despite these engineering works, problems of supply plagued the drought-exposed wetland system as Sydney’s population grew, encouraging a shift toward larger water storages more distal to the city, particularly the Upper Nepean scheme (Aird 1961). By 1886 the Botany Wetlands were virtually exhausted and were superseded as Sydney’s major water source, with management of the area given over to the newly created Board of Water Supply and Sewerage—the antecedent of the present management authority, Sydney Water Corporation—in 1888. From this time the Board leased land to industrial users with heavy demand for water, creating significant issues with pollution that persisted into the twentieth century. The establishment of golf courses on this degraded and formerly industrial landscape from 1904, with the last course established in 1960, preserved the ponds created under the Botany Swamps Supply Scheme once century earlier, but the creation and expansion of Sydney Airport from the 1940s significantly altered the southern reaches of the system. Parts of the wetland network were inscribed on the State Heritage Register (No 01317) in 2002, under section 170 of the *Heritage Act 1977* (NSW).

### Regulatory settings

Today, the Botany Wetlands contribute to a wide range of ecosystem services. In particular, the wetlands act as a connective habitat corridor and refuge for native flora and fauna including two threatened ecological communities—the ‘Eastern Suburbs Banksia Scrub’ (ESBS) and the ‘Sydney Freshwater Wetland’ (SFW). ESBS is listed as ‘critically endangered’ under Australia’s national biodiversity protection law, the *Environmental Protection and Biodiversity Conservation Act 1999* (Cth) (the EPBC Act), meaning “… it is facing an extremely high risk of extinction in the wild in the near future …” (section 182(2)). As a critically endangered ecological community, the ESBS must be protected in accordance with section 266B and an ‘approved conservation advice’ must be developed. A ‘conservation advice’ provides information about what could be done to stop decline or support recovery or a statement that there is nothing that can be done (section 266B). In addition, the ESBS is also subject to a ‘recovery plan’ (DEC 2004) that informs practical interventions by responsible parties, although recovery plans are not mandatory for threatened species under the federal legislation.

Parallel protections for the ESBS occur under the state government biodiversity conservation regime, the *Biodiversity Conservation Act 2016* (NSW) (BCAct), for New South Wales. Pursuant to this legislation the ESBS is listed also as *critically* endangered (BCAct, Schedule 2, Part 1). This categorization follows a review by the NSW Threatened Species Scientific Committee (2017) which identified an *extremely* high risk of extinction in the immediate future (section 4.4(1) and section 4.5(1) BCAct).

Sydney Freshwater Wetlands (SFW) are also protected through state regulation, which lists them as an endangered ecological community pursuant to Schedule 2 of the BCAct, meaning it is facing a very high risk of extinction in Australia in the near future (section  4.5, Division 2, Part 4 of the BCAct). This is despite the published view of the NSW Scientific Committee that SFW are “likely to become extinct in nature in New South Wales unless the circumstances and factors threatening its survival or evolutionary development cease to operate” (NSW Threatened Species Scientific Committee 2012).

The commitments to biodiversity conservation outlined above are operationalised through a Botany Wetlands Plan of Management (Sydney Water Corporation 1997, 2018). Both the ESBS and SFW are identified within the Plan of Management (PoM) with an assessment of the values, threats and opportunities for each (pp. 15–16). Within the PoMs actions is the call to “(i)dentify areas for potential expansion of existing ESBS” within the medium-term timeframe (estimated at the year 2020) and to “(c)ollaborate with key stakeholders to develop a Manual of Best Practice Management for Sydney Freshwater Wetlands” with a low priority timeframe (p. 24). Protection and regeneration actions are identified as ‘high priority’ (i.e.; ‘action required to avoid possible breach of legislation and prosecution’) in the PoM. This includes strategic expansion of the current 13 ha of ESBS communities and ‘regeneration’ to facilitate this.

These laws—as the framing legislation at the state and national level—and policies that enable actions on the ground, are merely part of a dense regulatory palimpsest that incorporates numerous legislative instruments ranging from the international to the local. There are at least 20 pieces of legislation or policy that concurrently shape and control the Botany Wetlands (Sydney Water Corporation 2018). In practice, this regulatory palimpsest acts to enforce an environmental narrative that is largely unquestioned or untested.

### Geohistorical method

The geohistorical component of this work was based on the analysis of sediment deposits that have accumulated over time within the ponds of the wetland corridor. Eight sediment cores from four pond sites (two replicate cores per site) were collected in April 2019 (Fig. 1) using a sliding hammer-and-collar device, deployed from a shallow-draft 3.21 m aluminium square nosed punt. Cores were capped and sealed, transferred to the laboratory and split vertically using a Geotek core splitter. Split cores were logged (Schnurrenberger et al. 2003) and photographed before being subjected to a series of analytical tests. Pond 5, as the most northerly of the remaining ponds, is the focus of this study.

Volume magnetic susceptibility was measured on the split cores with a Bartington MS3 meter and an MS2E sensor (Dearing 1994). Samples were measured in triplicate to constrain precision and the meter was calibrated to ambient magnetism before and after each sample measurement. Mineral particle size was measured with laser diffractometry using a Malvern Mastersizer 3000 with a wet dispersion unit. Samples were pre-treated to remove organic material using H_2_O_2_ and deflocculated using Na_4_P_2_O_7_ (Sperazza et al. 2004). Organic carbon was measured using the loss on ignition technique, with ignition at 550 °C for 2 h (Heiri et al. 2001).

Samples (1 cm^3^) for paleobotanical analysis were taken from core P5/04/19/A at 5 cm intervals between 5 and 110 cm depth (*n* = 24). Sample pre-treatment and analysis followed Faegri et al. (1989), and included deflocculation ((NaPO_3_)_6_), digestion of carbonate (HCl), silicates (HF) and cellulose (9:1 mixture of (CH_3_CO)_2_ and H_2_SO_4_). Samples were dehydrated (C_2_H_5_OH) and mounted (C_3_H_8_O_3_) for analysis at × 400–1000 times magnification. Pollen taxonomy is based on comparison with reference samples from vouchered specimens held at the National Herbarium of NSW, and by comparison with taxonomic databases (Australian Pollen and Spore Atlas^1^). Taxon abundance is expressed as relative abundance within the whole assemblage.

Age estimates for the sediment cores are based on a combination of lead and carbon radioisotope decay methods, and bio-stratigraphic dating. Samples of plant material (humified peat) were extracted from core P5/04/19A for radiocarbon dating, oven dried at 60 °C for 12 h, their dry mass measured, and subject to standard acid/alkali/acid pre-treatment prior to accelerator mass spectrometry. Samples for lead (^210^Pb) dating extracted from core P5/04/19/B, oven dried at 60 °C for 12 h then ground to a powder using a agate mortar and pestle. 2 g of each dried and ground sample was acid digested in a combination of HNO_3_, HCl and H_2_O_2_, prepared into an alpha source, and counted on an alpha spectrometer.

Biostratigraphic dating is based on the earliest (that is, stratigraphically lowest) appearance of pollen from exotic plants (typically the wind-pollinated exotic conifer *Pinus*). The timing of the first appearance of pine pollen in the sedimentary archive of the Sydney basin is heterogenous (see, for example, Fielding 1957; Mooney et al 2001; Black and Mooney 2006, 2007), but is taken here to be between 1840 and 1865 C.E. based on Dodson (1986), Conner and Thomas (2003), and Hamilton and Penny (2015).

## Results

### Stratigraphy and chronology

**Fig. 2:**
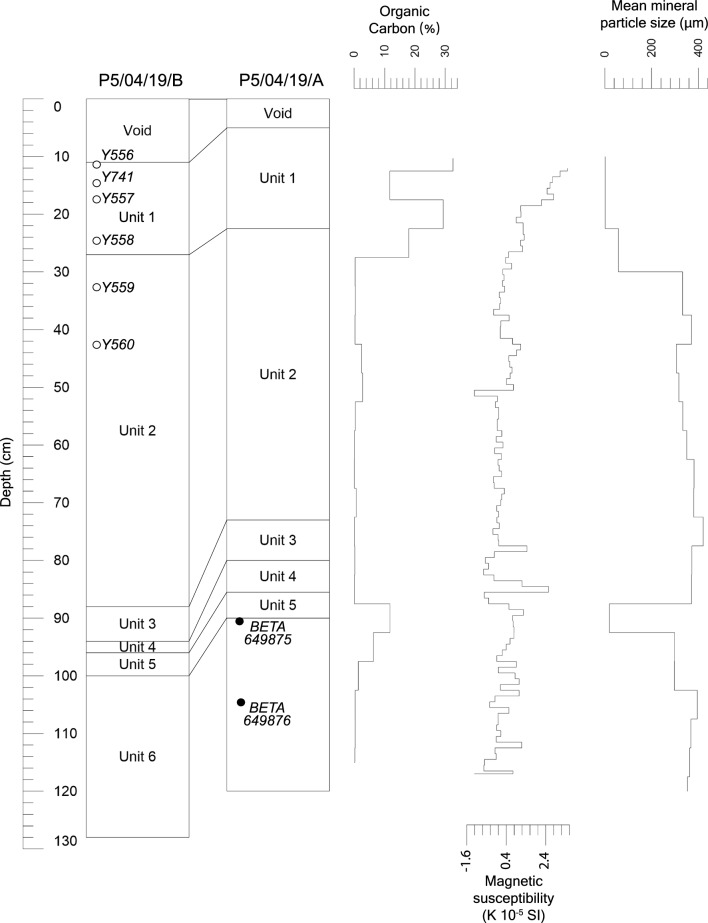
Stratigraphy of cores P5/04/19/A and P5/04/19/B. For unit descriptions see text. Lead dates (open circles, Table 2) and radiocarbon dates (closed circles, Table 1) are shown in stratigraphic context. Sedimentological data are for core P5/04/19/A only

Both cores from Pond 5 are comprised of the same six stratigraphic units, and these can be correlated directly between cores (Fig. 2). In core P5/04/19/A, Unit 1 (5.0–22.5 cm depth below lakebed; DBL) is a massively bedded black (2.5Y 2.5/1) humified peat or sapropel with an indistinct and irregular lower boundary. Unit 2 (22.5–73.0 cm DBL) is a light brownish grey sand (10YR 6/2) with few thin to very thin beds of black (2.5Y 2.5/1) humified peat above 30 cm DBL, with a sharp and sloping (c. 24° relative to the assumed bedding plane) lower boundary. Unit 3 (73.0–80.0 cm DBL) very dark grey (10YR 3/1) massively bedded sapropelic mud with a sharp and smooth lower boundary (c. 14° relative to the assumed bedding plane). Unit 4 (80.0–85.5 cm DBL) is a black (2.5Y 2.5/1) massively bedded organic sandy loam with an indistinct lower boundary. Unit 5 (85.5–95.0 cm DBL) is a very dark grey (2.5Y 3/1) organic sand with an indistinct and irregular lower boundary. Unit 6, the stratigraphically lowest unit (95.0–120.0 cm DBL), is a grey (2.5Y 5/1) massively bedded sand. The same sequence of materials occurs in P5/04/19/B, with slightly different boundary depths (Fig. 2). The thin beds of black humified peat or sapropels observed in Unit 2 in Core A were more strongly developed in upper part of Unit 2 in core B above 50 cm DBL, forming thin (3–10 cm thick following Schnurrenberger et al. 2003) to very thin (1–3 cm thick) beds, particularly at 32–32.5, 33–34, 40.5–44, 49–50, 59.5–60 and 78–79 cm DBL. Gravel and granule-sized mineral clasts were also observed as a discrete very thin bed associated with the organic bed at 78–79 cm DBL in Unit 2 of core B.

Radiocarbon results (Table 1) from Core A indicate that the organic sand (Units 5 and 6) at the base of the core was deposited before the fifteenth century C.E. This places the formation of the swamp more than 570 years before the ponds were dammed to provide fresh water for the colony of Sydney and, in fact, nearly 500 years before the arrival of Europeans in Kamay Botany Bay. While this result demonstrates unequivocally that the modern ponds are contemporary remnants of natural waterbodies with much greater antiquity, the deposits are young in geomorphic terms. We might infer from this that large floods within the drainage system have episodically scoured accumulated alluvial sediment, thus removing any evidence of older deposits from this site. Vigorous erosion of the site due to flooding, including partial destruction of the pond dams, was reported in the late 1860s and early 1870s (Thorpe et al. 1991), during a particularly wet period for south-eastern Australia (Ashcroft et al. 2016).

**Table 1:** Radiocarbon results for core P5/04/19/A. Radiocarbon ages calibrated using Calib 8.2 (Stuiver and Reimer 1993) using the SHCal20 dataset (Hogg et al. 2020). Proportion of the probability distribution for multiple intersections with the calibration curve given in square brackets

Lab. code	Depth (cm)	14C years BP ± 1 $\sigma$	Cal. Yrs BP 2 $\sigma$ 95.4% prob	Cal Yrs CE	Median Prob. CE
BETA649875	90–91	520 ± 30	496–542 [1]	1408–1454 [1]	1433
BETA649876	104–105	750 ± 30	565–596 [0.311] 631–682 [0.648] 705–718 [0.041]	1232–1245 [0.041] 1268–1319 [0.648] 1354–1385 [0.311]	1294

The unsupported ^210^Pb activity (Table 2) was quite high for this core, meaning that the chronological models were able to reach older ages (between 100 and 150 years ago). Both the constant flux constant sedimentation (CFCS) and constant rate of supply (CRS) models used to calculate ages were in good agreement in the upper part of the sequence due to a monotonic decrease in total ^210^Pb with depth, although the age of the last depth may be somewhat overestimated because of the higher variability of the unsupported ^210^Pb activity in the last three depths analysed (24, 32, 42 cm DBL). The CRS model indicates that stratigraphic Unit 2, a light brownish grey sand, was deposited from the mid-nineteenth century C.E. to the late twentieth century C.E. Unit 1, a humified peat, was deposited from the early 1990s, and may reflect an increase in accommodation space associated with the reconfiguration of the system’s outflow to accommodate a new runway for Sydney Airport. The CFCS model is not used in the interpretation below as its assumptions regarding the stability of sedimentation rates through the sequence cannot be supported given the stratigraphy described above.

**Table 2:** Lead dating results for core P5/04/19/B

Lab code	Depth (cm)	Supported ^210^Pb (Bq/kg)	Unsupported ^210^Pb (Bq/kg)	Calculated CRS ages (years)	Calculated CFCS ages (years)	CRS mass accumulation rates (g/cm^2^/year)
Y556	11–12	20 ± 2	258 ± 12	7 ± 2	7 ± 1	0.170 ± 0.010
Y741	14–15	20 ± 2	225 ± 11	15 ± 1	14 ± 3	0.151 ± 0.007
Y557	17–18	21 ± 2	130 ± 7	22 ± 2	21 ± 4	0.207 ± 0.011
Y558	24–25	17 ± 2	51 ± 3	28 ± 2	29 ± 5	0.449 ± 0.021
Y559	32–33	1 ± 0	73 ± 3	56 ± 2	65 ± 12	0.129 ± 0.007
Y560	42–43	4 ± 0	4 ± 1	149 ± 8	126 ± 22	0.116 ± 0.029

### Palynology and biostratigraphy

Pollen counts for Core A varied between a maximum of 326 individuals (90 cm depth) and a minimum of 3 (110 cm depth), with an average count of 141 ± 99 individuals. Of the 24 samples analysed, 9 (40–70 and 115–120 cm depth) yielded no microfossils, coincident with the deposition of stratigraphic units 2 and 6.

The lack of pollen preservation in samples between 40 and 70 cm depth (Unit 2), and below 115 cm depth (Unit 6), is associated with the deposition of medium-coarse sand within the pond. While Unit 6 is likely derived from the underlying Botany Sands Formation and is presumably heavily oxidised, Unit 2 was deposited much more recently. The stratigraphically lowest ^210^Pb age from core B (Y560, 42.5 cm DBL: Table 2) gives an age of 1870 C.E., shortly after the damming of the ponds as part of the BSWSS in 1866/67 C.E. It is possible that the lower part of Unit 2 was deposited rapidly as a consequence of dam construction both above and below Pond 5 (Larcombe 1963). The dams were timber piles, faced with timber, and filled with sand presumably excavated from the surrounding rolling dunes (Thorpe et al. 1991). This would have mobilised large volumes of oxidised sand that settled rapidly in the ponds. The thin bed of coarse clastic material observed at 78–79 cm DBL in Core B, toward the base of Unit 2, is suggestive of a discrete high energy event. Reduced sediment accumulation rates after 1870 are implied by gradually increasing organic carbon and fine-grained mineral sediment in Unit 2 in both cores (Fig. 2).

Pollen from exotic trees is first observed at 90 cm depth—a single specimen of pine pollen—with oak, beach and pine occurring at and then continuously above 80 cm depth (Fig. 4). The occurrence of pine at 90 cm depth can be disregarded as contamination as a radiocarbon date at that depth (BETA649875; Table 1) indicates a probable median age of 1433 C.E, long before the introduction and spread of pine through the Sydney basin in the early-mid nineteenth century. The consistent appearance of exotic plants from 80 cm depth is more convincing, but demands either a rapid decrease in sedimentation rate above 90 cm depth, or a stratigraphic discontinuity most probably at the sharp lower boundary between units 3 and 4 at 80 cm depth in core A and 94 cm depth in core B. *Ficus* pollen is first recorded in the mid-twentieth century C.E. (30 cm depth, top of Unit 2), nearly one century after *F. macrophylla* and *F. rubiginosa* were first planted as ornamental trees in 1869 (Thorpe et al. 1991).

**Fig. 3:**
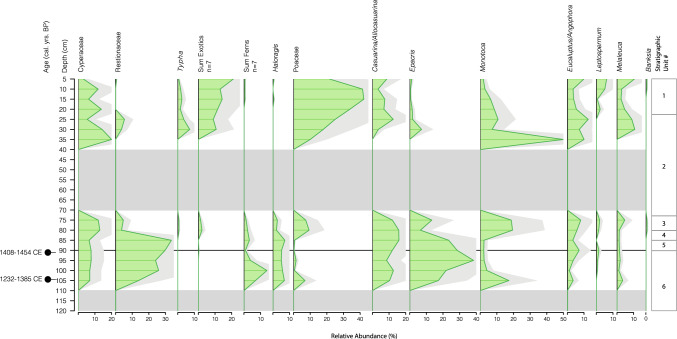
Pollen and spore data for selected taxa, plotted against depth, for core P5/04/19/A. Taxa are expressed as relative abundance of the total pollen assemblage, with a 2 × exaggeration (pale grey shading). Only those taxa with a relative abundance > 10% are shown. Grey zones 40–70 cm and > 110 cm represents samples with no pollen preservation, coincident with the deposition of sand beds. The black line represents the first appearance of pollen from exotic plants

**Fig. 4:**
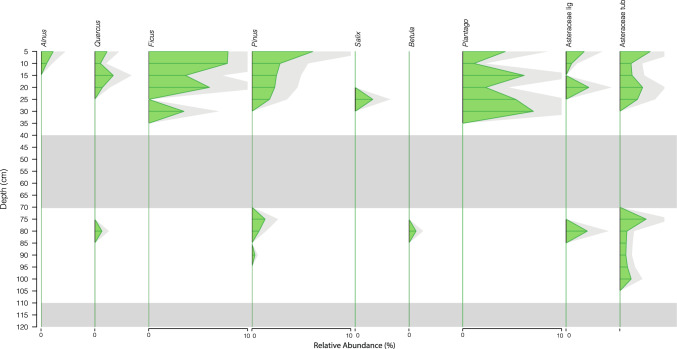
Pollen and spore data for exotic taxa only, plotted against depth, for core P5/04/19/A. Taxa are expressed as relative abundance of the total pollen assemblage, with a 2 × exaggeration (pale grey shading). Grey zones 40–70 cm and > 110 cm represents samples with no pollen preservation, coincident with the deposition of sand beds

Our pollen results (Figs. 3 and 4) indicate that the vegetation proximal to Pond 5 has changed markedly since the end of the thirteenth century C.E. Notably, a distinctive community type dominated by the heath plants *Epacris* and *Monotoca,* rushes (Restionaceae), and the herb *Haloragis* appears to have been common at the site until the nineteenth century, at the putative discontinuity between Units 3 and 4. This community type was replaced rapidly by a hybridised ecological community characterised by sedges (Cyperaceae), grasses (Poaceae), exotic plants, and genera from the Myrtaceae family (particularly *Melaleuca*, or paperbark). This process appears to have been ongoing during the middle nineteenth century and was largely complete by 1870 once the dams were emplaced and the system dramatically modified. This accords well with the historical information, which indicates very little disruption to the swamp vegetation as late as 1846 (Thorpe et al. 1991, p. 30), but that by 1869 very little of the original swamp vegetation remained (ibid, pp. 33,34).

Of the dominant taxa of the ESBS (Perkins et al. 2012) only *Monotoca* (possibly derived from *M. elliptica* and *M. scoparia*) appears in the pollen record prior to the major modifications of the wetland system in the mid nineteenth century. *Banksia*, in particular, occurs in only four samples and in all cases represents less than 1% of the total pollen assemblage. It is not present at all below 80 cm, prior to the first indications of European presence in the landscape. The dominant woodland taxon revealed in the pollen data, prior to the modification of the system, is *Casuarina/Allocasuarina* (possibly derived from Scrub She-oak, *Allocasuarina distyla*), with low but consistent representation of pollen from *Eucalyptus/Angophora* type, *Melaleuca* and *Leptospermum* types, the latter likely derived from the fringes of wetland communities where these taxa are more common.

Equally, the current Sydney Freshwater Wetlands (SFW) is dominated by Cyperaceae (9 species from 5 genera). Cyperaceae pollen is present throughout the record, but increases slightly in abundance after the 1870s. However, our data indicate that, prior to the re-organisation of the system for Sydney’s water supply, the dominant wetland plants belonged to the Restionaceae family. Only three members of that family (*Baloskion tetraphyllum*, *Emphodisma minus* and *Leptocarpus tenax*) are currently listed as part of the SWF assemblage. Pollen from grasses (Poaceae), which was a minor component of the assemblage prior to the 1870s, increases markedly after the emplacement of the dams to become the dominant herbaceous pollen type in the most recent sediment. Ferns and *Haloragis/Gonocarpus* type (possibly *Gonocarpus teucrioides*, part of the ESBS assemblage) were also common elements that appear to have been severely disadvantaged by the disruption of the system.

## Discussion

Our analysis indicates that high-value threatened ecosystems that are protected in law and policy with the Botany Wetlands are compositionally different from the ‘authentic’ native vegetation that occurred on the site from at least the thirteenth century C.E. to its extirpation during the nineteenth century. We note with some interest that the floristic changes described here are nearly identical to those described by Hamilton et al. (2015) from Lachlan Swamp, in what is now Centennial Park. Lachlan Swamp, some 4 km to the north of our site, is a natural spring that fed the upper reaches of the Botany Wetlands. There, the *Epacris*-Restionaceae dominated heath communities, which had been present on the site for at least 4,000 years, persisted into last decade of the nineteenth century before they were progressively replaced by the ornamental plantings of broad-leaved paperbark (*Melaleuca quinquenervia*) that exist there today. Taken together, these data imply a locally extensive coastal heath community that had existed in place through the Upper Holocene at least, but which is no longer represented in the extant flora. More pointedly, this ‘authentic’ vegetation community is specifically excluded from the landscape by a dense regulatory overburden that valorises ‘endangered’ vegetation communities that are, in fact, the product of European land use practices.

The stark contrast between the original native vegetation and the threatened native vegetation conserved within the Botany Wetlands raises important issues. As described earlier, both the ESBS and SFW are afforded protections through the *Environmental Protection and Biodiversity Conservation Act 1999* (C’th) and the *Biodiversity Conservation Act 2016* (NSW), and these protections are operationalised through Sydney Water’s Plan of Management (Sydney Water Corporation 2018) and through the subordinate Environmental Management Plans enacted by the three golf clubs that currently lease the majority of the wetland corridor. For example, the Eastlakes Golf Club’s Environmental Management Plan (EMP; Narla Environmental 2019) describes ESBS as ‘remnant’ vegetation, reflecting the ‘authentic native’ narrative embedded in the regulatory framework in which it exists. Drawing heavily on the New South Wales government’s ESBS Recovery Plan (DEC 2004), the EMP stipulates the replanting and expansion of ESBS and advises that re-constituted ESBS stands enjoy the same legislative protections as ‘remnant stands’. This is the precise mechanism by which ‘on the ground’ interventions create and legitimise an environmental narrative in the material fabric of landscape.

The Botany Wetlands are, at once, a water supply, a hunting ground, an industrial drain, a site for recreation and a critically important conservation corridor. We have demonstrated here that its functions and the composition of its plant communities are a product of its history and a dense regulatory network that acts to enforce a specific, largely unquestioned, environmental narrative. Adherence to that narrative effectively limits the range of adaptive choices available to management authorities when confronted by rapid and systemic change. Management practice is shackled to a ‘scientific’ designation of nativeness and its conservation values rather than being free to focus on how best to hybridise ecological communities to achieve sustainable environmental services. The chronic stress of climate change, and the attendant acute disruption of our weather, demands a greater focus on the resilience of environmental services from urban green spaces over centuries, rather than until the development of the next Plan of Management. In this context, Gould’s (1998) *genius loci* becomes important, not because native plants are better by definition, but because adaptation in place over long time periods demonstrates resilience to change. In this context, we argue that resilient plant communities that can be defined by the self-organised, dynamically stable assemblage of species that can only be determined by considering the long time scales over which self-organisation becomes apparent. The persistence of a dynamically stable plant community over centuries or millennia necessarily demonstrates resilience to high-amplitude/low-frequency environmental stressors that have occurred over those time periods. Evidence of the long term persistence in place demonstrated by plant communities themselves—letting the plants speak—offers opportunities for environmental managers to mimic those communities for greater resilience to projected change.

Functional traits that support ecosystem services—particularly those relevant to urban environments—might be maintained in ‘crappy’ hybridised socio-ecological systems as well as they can in ‘natural’ systems. However, the prospect of maintaining those services over time in the context of climate change and other systemic stressors (population growth, pollution, biodiversity loss, etc.) appear dim or, at least, are brighter if we can mimic the composition and distribution of natural systems that we know have endured climatic perturbation and intensive fire-management over thousands of years. There is nothing special about nativeness, per se, but it does offer us the best opportunity of building resilience into green and blue spaces that might secure sustainable urban environments into the future. For example, our data suggest that greater resilience to low-frequency but high-amplitude perturbation, including but not restricted to climatic variability, can be achieved by increasing the abundance of some taxa (particularly several genera of the Epacridaceae and Restionaceae) at the expense of others currently listed as part of the ‘authentic’ remnant native ecosystems (such as genera of the Cyperaceae as part of the SWF community, or Proteaceae as part of the ESBS).

## Conclusions

Our attention to geohistorical data in the context of contemporary management frameworks exposes some important tensions—chiefly between the assumptions that underpin the protection of threatened ecological communities and the actual ecological history of the site. Specifically, our data indicate that the original vegetation communities in and around the wetland were compositionally different from the contemporary flora, and that neither bear any close resemblance, floristically, to the critically endangered ecological communities whose conservation within the wetland corridor is enforced through law and management practice.

The practice of environmental management—those on-the-ground actions that make and re-make our material landscapes—is enabled through law, either directly or via policy and normative institutional practices. Legal geography—explicitly concerned with the co-constitution of people–place-law dynamics—has much to offer in exposing the pathways by which environmental imaginaries become encoded and legitimised in the material landscape (Gillespie 2015). The work of legal geography, in a vast variety of settings, exposes the ways in which laws (and indeed ‘lores’; see, for example, Gillespie 2018) emerge in reaction to, and create, people/place dynamics. Delaney (2014, 2015, 2016) identified the ‘promise to open up a space for critical *legal* physical—or environmental—geographies.” (2016, p.6). We contribute to this endeavour, for we continue to argue against the de-coupling or abstraction of law from the materialities of landscape and resources—the ‘de-physicalisation’ of socio-legal processes (Graham 2010; Bartel and Graham 2015; Gillespie 2020; O’Donnell et al. 2020; Bartel and Carter 2021). This scholarship allows us to appreciate the role of institutions and governance structures in creating and, at times, freezing places.

Our analysis demonstrates that, in this case at least, the landscapes we conserve spring entirely from unstated assumptions about ‘naturalness’ and the operation of law and, given time, are legitimised by “enculturated attachments to familiar landscapes” (Trigger and Head 2010) and by regulatory structures and practices that continually make and re-make these imaginaries. By specifically planting some types of plants and excluding others, for example, we make an ecosystem that then becomes—almost by dint of simply being there—credible, defensible and even desirable. Geohistorical data might provide a pathway back to ecological ‘authenticity’ (Dietl and Flessa 2011) and, perhaps, improved environmental services, but also demonstrate empirically how far contemporary ecosystems may have drifted from that normative ideal of environmental authenticity. These approaches, then, may also undermine the credibility of managed and highly-valued urban green and blue spaces. As Richardson says, “the difficulty with restoring layered geographies is that revival of natural heritage may clash with respect for the culture heritage in modified landscapes. … There is no easy solution to this tension” (Richardson 2017, p. 156). The point is we need to go beyond entrenched and largely fruitless debates about ecological ‘belonging’ or ‘authenticity’ and embrace the opportunities of crappy, degraded or otherwise hybridised landscapes—to render their complexity down to what arrangements can best secure a resilient, adaptable and sustainable future.

## Footnotes

1 http://apsa.anu.edu.au.
